# Current surveillance in the Netherlands for urothelial carcinoma in lynch syndrome

**DOI:** 10.1007/s10689-025-00503-6

**Published:** 2025-10-18

**Authors:** B. H. J. Doornweerd, R. R. Kikkert, J. J. Koornstra, B. W. G. van Rhijn, E. J. van Gennep, A. M. J. Langers, I. J. de Jong, M. E. van Leerdam

**Affiliations:** 1 Department of Urology, University Medical Center Groningen, University of Groningen, Groningen, The Netherlands; 2 Department of Gastroenterology and Hepatology, University Medical Center Groningen, University of Groningen, Groningen, The Netherlands; 3 Department of Gastrointestinal Oncology, Netherlands Cancer Institute, Amsterdam, The Netherlands; 4 Department of Urology, Netherlands Cancer Institute, Amsterdam, The Netherlands; 5 Department of Gastroenterology and Hepatology, Leiden University Medical Center, Leiden, The Netherlands; 6 Department of Urology, Leiden University Medical Center, Leiden, The Netherlands; 7 Netherlands Foundation for Detection of Hereditary Tumors, Leiden, The Netherlands

**Keywords:** Lynch syndrome, Urothelial carcinoma, Surveillance, Urinary cytology

## Abstract

Lynch syndrome (LS) is a hereditary cancer predisposition syndrome caused by a pathogenic germline variant in one of the mismatch repair (MMR) genes. There is a lack of evidence supporting surveillance for urothelial carcinoma (UC) in LS, however several surveillance strategies have been proposed in recent years. This has led to a variety of practices. This study reports on the current practice for UC surveillance in LS in the Netherlands. Individuals with LS from two university hospitals and a large comprehensive cancer center were selected. Data on UC surveillance and UC diagnoses were recorded. Cumulative incidence was calculated. Of 235 individuals with LS, 40% underwent UC surveillance. Individuals with a pathogenic *MSH2* variant were under surveillance significantly more often than those with other pathogenic variants. Of the individuals who underwent surveillance 10% had annual testing and continued surveillance up until the last known follow up date. None of the surveillance tests led to a UC diagnosis. Of eight patients with UC, two were under active surveillance, but were diagnosed due to macroscopic hematuria after normal cytology results. Cumulative UC incidence was 9% at age 75. Currently, most individuals with LS in this cohort are not part of a surveillance program for UC. Cumulative UC risk is high, up to 9%, and none of the UC were detected by surveillance. Given the lack of evidence that a suitable surveillance test is available, we do not recommend surveillance for UC.

## Introduction

Lynch syndrome (LS) is a hereditary cancer predisposition syndrome caused by a pathogenic germline variant in one of the mismatch repair (MMR) genes [1]. Individuals with LS have an increased lifetime risk for various cancers, including colorectal and endometrial cancer [2, 3]. Those with a proven pathogenic MMR variant are advised to undergo surveillance for colorectal and endometrial cancer. First-degree relatives, who are up to 50% risk-carriers, are advised to either undergo genetic testing, or undergo surveillance as advised for those with a proven pathogenic variant. The goal of surveillance is to improve survival by diagnosing cancer in an early stage to make an early treatment possible [4, 5].

In the Netherlands, individuals with LS are seen biennially by a gastroenterologist for surveillance colonoscopy from the age of 25 years, and annually for gynecological surveillance for females 40–60 years of age. The gastroenterologist coordinates most of the care for these individuals. Urothelial carcinoma (UC) of the bladder and upper urinary tract are part of the tumor spectrum in LS, especially in those with a pathogenic *MSH2* variant [6–8]. High-grade UC is an aggressive disease with potential cancer-related mortality in LS [9, 10]. While evidence supporting surveillance for UC in LS is limited, several surveillance strategies have been suggested in recent years, mostly based on expert opinion [11]. Since 2015 surveillance for UC has not been recommended by the Dutch guidelines on familial and hereditary cancer due to the lack of proven benefit, following the recommendation of a group of European experts [12].

We hypothesize that there is significant variability in current practices among gastroenterologists regarding UC surveillance and the referral of LS individuals to a urologist. This study aims to report on the cumulative risk of UC in a large LS cohort. Furthermore, we evaluate how often surveillance for UC has been performed, what tests have been used, and how many cases of UC were detected through surveillance in individuals with LS. Through this, we aim to assess the current Dutch surveillance practices and the effectiveness of surveillance over the past 30 years in LS individuals.

## Patients and methods

All individuals with LS under surveillance by a gastroenterologist at two university hospitals and a comprehensive cancer center in the Netherlands and registered at the Netherlands Foundation for Detection of Hereditary Tumors (StOET) were included in this study. Following a LS diagnosis in the Netherlands, families are invited to register at the nationwide registry of the StOET [13].

Individuals with LS were defined as those with a pathogenic MMR variant. Electronic patient records were used to collect data on patient characteristics, type of pathogenic variant (*MLH1, MSH2 including silencing through EPCAM, MSH6 and PMS2*) oncological history, family history of urological malignancies, and performed surveillance tests: urinary dipstick for hematuria, urinary cytology, kidney ultrasound, and other tests (for example computed tomography). For urinary dipstick, any result with (microscopic) hematuria was considered suspicious for UC. For kidney ultrasound, hydronephrosis or a renal mass was considered suspicious, while urinary cytology was considered suspicious if atypical or malignant cells were detected. In case of UC diagnosis data on the trigger that led to the diagnosis (surveillance or symptoms), age at time of diagnosis, staging, grading, treatment and outcome were collected.

Cumulative incidence at age 75 for UC was estimated using the life table method. For each individual (N = 235), the age (in years) at cancer diagnosis or the last known age to be cancer-free was recorded. Subjects were considered to have undergone surveillance for UC if they received any form of urinary tract surveillance. Surveillance was considered discontinued if no surveillance tests were performed in the year prior to the UC diagnosis or the last known contact date. Periodic surveillance was considered structured if surveillance tests are performed at least once a year.

The study was approved by the medical ethics committee (University Medical Center Groningen research registry number: 202000463). Pseudo-anonymized study data were collected and managed using REDCap electronic data capture tools hosted at University Medical Center Groningen [14, 15]. For data analysis IBM SPSS Statistics for Windows, Version 28.0 (IBM Corp., Armonk, N.Y., USA) was used.

## Results

A total of 235 individuals were included, with a median age of 57 years (range: 24–90 years). Of these, 34% were male, and 14.9% were former or current smokers. The first registered contact with a health care provider in one of the university hospitals or comprehensive cancer center was in January 1992, and the last registered contact was in January 2022. The mean follow-up time was 150 months (standard deviation = 83 months). Patient characteristics are shown in Table 1.

**Table 1:** Characteristics of 235 individuals with Lynch syndrome

	Nr (%)
*Sex*	
Female	156 (66,4)
Male	79 (33,6)
Median age in years (range)	57 (24–90)
*MMR pathogenic variant*	
MLH1	55 (23,4)
MSH2*	60 (25,5)
MSH6	89 (37,9)
PMS2	25 (10,6)
missing	6 (2,6)
*History of smoking*	
Yes	35 (14,9)
No	136 (57,9)
missing	64 (27,2)
*Family history of UC*	
Yes	31 (13,2)
No	155 (66,0)
missing	49 (20,9)
*UC surveillance*	
Yes	95 (40,4)
No	140 (59,6)
*History of other LS associated malignancy*	
Yes	67 (28,5)
No	166 (70,6)
Unknown	2 (0,9)

^*^Including silencing through EPCAM (n = 7)

### Surveillance

A total of 95 individuals (40%) underwent one or more tests for urinary tract surveillance. Among individuals with a pathogenic *MSH2* variant, 62% underwent one or more rounds of surveillance, compared to 35% in individuals with other pathogenic variants (*p* < 0.01). A total of 66/95 (69%) discontinued surveillance. In 9 individuals (9%), information on the continuation of surveillance is missing, while in 20 individuals (21%) surveillance was continued until the end of the follow-up period. Of these 20 individuals, 10 (50%) underwent structured surveillance with testing at least once a year. A total of 140 patients (60%) did not undergo any surveillance for UC.

### Surveillance tests

Of the 95 individuals who underwent UC surveillance, 43 (45%) had urinalysis, 50 (53%) had a kidney ultrasound, and 83 (87%) had urinary cytology. In 20 individuals (21%), other tests were performed, mostly CT scans for the follow-up of other malignancies and not primarily for UC surveillance. None of these tests led to a UC diagnosis.

### Urinalysis

In 43 individuals, 89 urinalyses were performed for surveillance purposes (median per subject 1, range 1–5). Of these, 15 (17%) had non-visible hematuria, 71 (80%) had normal results, and the results of 3 tests (3%) were missing. None of the abnormal results led to a UC diagnosis.

### Kidney ultrasound

In 50 individuals, 105 kidney ultrasounds were performed (median per subject 3, range 1–14). Of these, 99 were normal, 2 showed hydronephrosis; in both cases, no UC was diagnosed. The results of 4 ultrasounds were missing.

### Cytology

A total of 373 cytologic examinations of the urine were performed for surveillance purposes in 79 individuals, with a median of 3 examinations per subject (range 1–27). Nineteen cytology reports (5.1%) were abnormal in 10 individuals (median 1.5; range 1–3). None of these individuals were diagnosed with UC. A total of 345 (92.5%) cytology reports were unsuspicious, and in nine examinations (2.4%) the results were not available for this analysis.

### Urinary tract cancer

A total of eight patients were diagnosed with UC including 4 patients with a pathogenic *MSH2* variant, 3 patients with a pathogenic *MLH1* variant and 1 patient with a pathogenic *MSH6* variant (Table 2). Median age at diagnosis was 59 years (range 40–66 years). Estimated incidence for UC was 9% at age 75. Two patients had a positive family history of UC, both carrying a pathogenic *MSH2* variant. One patient had a family history of renal cell carcinoma. Three patients had a negative family history for urological malignancies, and in two patients, family history was not documented.

**Table 2:** Urothelial carcinoma diagnoses

Location cancer	Age at diagnosis	Sex	MMR PV	Smoker	Mode of detection	Years since first visit*	Stage	Grade	(Prior) Surveillance for UC
Ureter	60	Female	*MSH6*	No	Missing	12	TaNxMx	2	No
Ureter	66	Female	*MSH2*	No	Pain, mass	25	TxN2M1	Missing	Yes (discontinued)
Bladder	59	Male	*MSH2*	Yes	Coincidental finding in staging for colon carcinoma	12	T2N2M0	HG	Yes (discontinued)
Ureter	59	Female	*MLH1*	Yes	Hematuria	16	T3N0M0	HG	Yes (discontinued)
Bladder	61	Male	*MLH1*	Missing	Missing	13	TaN0M0	1	Yes (discontinued)
Ureter	40	Male	*MSH2*	No	Hematuria	0	T1NxMx	3	No
Bladder	61	Male	*MLH1*	No	Hematuria	1	TaN0M0	1	Yes (cytology normal)
Bladder	56	Male	*MSH2*	No	Hematuria	13	T2N0M0	3	Yes (cytology normal)

MMR PV, pathogenic mismatch repair gene variant; HG, high grade; UC, urothelial carcinoma

^*^Either with the gastroenterologist or other health care professional

Four of the eight patients with UC (50%) presented with macroscopic hematuria. One patient presented with a palpable mass and abdominal pain, one patient was diagnosed with UC as a coincidental finding on CT (for colon carcinoma) and in 2/8 (25%) the method of diagnosis was not clearly documented, but no surveillance was performed at that time.

Of all eight patients with UC, two (25%) did not undergo UC surveillance, while four (50%) had been under surveillance but discontinued it (3, 10, 13, and 19 years before UC diagnosis). Two patients (25%) were diagnosed due to symptomatic hematuria, despite being under active surveillance. Both had normal cytology results one year prior to their diagnosis.

Among the eight UC patients, four patients (50%) were diagnosed with bladder cancer, while the other four (50%) had upper urinary tract cancer. One patient was diagnosed with synchronous distant metastases and was treated palliatively, while the other seven patients were treated with curative intent.

## Discussion

A range of recommendations on surveillance for UC in LS has been given in the past years, even though there is lack of evidence supporting any of these recommendations. In this study we showed that 40% of individuals with LS in a large cohort underwent surveillance for UC. Estimated cumulative incidence at age 75 of UC is 9%. None of the reported UC’s were detected by surveillance.

### Execution of surveillance

Our results show that surveillance is performed more frequently in individuals with a pathogenic *MSH2* variant than in those with other pathogenic variants. This is consistent with publications recommending surveillance for individuals in this highest-risk category [16, 17]. Only 10% of the individuals who underwent surveillance had at least annual testing and continued surveillance up until the last known follow up date. In the majority of individuals surveillance tests are performed without a predefined schedule. This may be due to changes in guideline recommendations, a lack of a structured protocol, or the failure to follow available recommendations. Also personal preferences of individuals with LS may play a role in either continuing or discontinuing surveillance.

In none of the UC patients surveillance led to the diagnosis, despite 6 out of 8 UC patients being enrolled in some sort of a surveillance program for UC. In two UC patients who were part of a surveillance program urinary cytology was normal one year before diagnosis and surveillance failed either due to the low sensitivity of the test or the short lead time of UC. In four patients, surveillance may have failed due to early discontinuation. Although it cannot be stated with certainty that the continuation of surveillance would have led to an early diagnosis, its discontinuation may have resulted in a missed opportunity. At least five patients with UC presented with symptoms. Failing to diagnose these patients while asymptomatic may have led to a more advanced stage at diagnosis. This study was not conducted to report on the test properties of surveillance tests, but low sensitivity of all performed tests is likely.

### Methods of UC surveillance

A total of 97 patients underwent UC surveillance and none of the surveillance tests has led to a UC diagnosis. Moreover, none of the UC diagnosis was made because of surveillance. Urinary cytology has been used most frequently in this cohort. In a Danish study the sensitivity of urinary cytology for UC in asymptomatic individuals with LS was found to be 29%, with a specificity of 96% [18]. In our study only two patients with UC were in an active surveillance protocol with urinary cytology. Both had negative results prior to the diagnosis and became symptomatic.

In none of the reported UC cases surveillance ultrasound was performed prior to the diagnosis, making it impossible to assess the sensitivity of ultrasound in our cohort. Reported sensitivity for upper tract UC in individuals with hematuria varies greatly (46–100%) [19–22], but generally, ultrasound is considered unsuitable for imaging the ureters [23].

The early detection of microscopic hematuria may lead to an earlier diagnosis in patients with high-grade UC of the bladder [24], but it is associated with a high false-positive rate. Our results show a false-positive rate of 17%; true positives and therefore sensitivity could not be determined because urinalysis was not performed in the UC cases included in this report.

Lonati et al. recommend bi-annual imaging by ultrasound alternating with CT urography in individuals with a pathogenic *MSH2* variant or those with a positive family history of UC [11]. None of the individuals in our cohort underwent such a protocol, despite one coincidental finding on CT. The benefits of frequent CT scans should be weighed against the potential risks of radiation exposure and costs. Prospective data are needed to support such a recommendation.

### Challenges of UC surveillance in LS

The lifetime cumulative incidence of UC of 9% in this report supports that surveillance is appropriate, provided that suitable tests are available. Our data do not allow us to comment on the best tests to perform, but it is likely that the surveillance programs failed due to low accuracy, unstructured and/or discontinued testing. Given the lack of evidence that a suitable surveillance test is available, we do not recommend surveillance for UC. Because of the high lifetime risk for UC, further research into surveillance test is necessary focusing on accuracy of tests, the impact on survival and the cost-effectiveness of such surveillance programs.

## Conclusions

Although individuals with LS have a high lifetime risk of developing UC of the bladder and upper urinary tract, there is no unambiguous guideline on surveillance for UC in these individuals. Currently, most individuals are not part of a surveillance program for UC. Among those who have undergone at least one surveillance test, the majority either discontinue surveillance or undergo testing in an unstructured manner. In our prospective cohort study none of the UC was diagnosed by surveillance.

## Data Availability

No datasets were generated or analysed during the current study.
